# ERCC6L2-related disease: a novel entity of bone marrow failure disorder with high risk of clonal evolution

**DOI:** 10.1007/s00277-023-05128-2

**Published:** 2023-02-15

**Authors:** Francesco Baccelli, Davide Leardini, Sara Cerasi, Daria Messelodi, Salvatore Nicola Bertuccio, Riccardo Masetti

**Affiliations:** 1 Pediatric Oncology and Hematology Unit “Lalla Seràgnoli”, IRCCS Azienda Ospedaliero-Universitaria di Bologna, Via Giuseppe Massarenti, 11, 40138 Bologna, Italy; 2 Department of Medical and Surgical Sciences, University of Bologna, Bologna, Italy

**Keywords:** Myelodysplastic syndrome, Acute myeloid leukemia, ERCC6L2, Hematopoietic stem cell transplantation, Cancer predisposition

## Abstract

ERCC excision repair 6 like 2 (*ERCC6L2*) gene encodes for different helicase-like protein members of the Snf2 family involved in transcription-coupled nucleotide excision repair and in cell proliferation. Germline homozygous mutations in children and adults predispose to a peculiar bone marrow failure phenotype characterized by mild hematological alterations with a high risk of developing acute myeloid leukemia. The outcome for patients with leukemia progression is dismal while patients undergoing hematopoietic stem cell transplantation in the early stage have better outcomes. The ERCC6L2-related hematological disease presents a high penetrance, posing important questions regarding the treatment strategies and possible preemptive approaches. This review describes the biological function of ERCC6L2 and the clinical manifestations of the associated disease, trying to focus on the unsolved clinical questions.

**Supplementary Information:**

The online version contains supplementary material available at 10.1007/s00277-023-05128-2.

## Introduction

Over the last few years, the broad application of large-scale genomic sequencing has revealed a large variety of germline variants associated with inherited bone marrow failure (BMF) syndromes [1–5]. These variants are associated with hematological and often extra hematological clinical features, defining complex multisystemic syndromes [6–8]. Some of these disorders present a variable risk of clonal evolution and progression to myelodysplastic syndrome (MDS) and acute myeloid leukemia (AML) [9]. The biological study of these variants is leading to a deeper understanding of the genetic origin and pathogenesis of these diseases, eliciting new diagnostic categorization of BMF [10, 11]. The different genetic driving mutations underpinning these entities result in highly different hematological phenotype with a different temporal evolution which is often difficult to predict. These considerations pose challenging questions to clinicians, regarding the therapeutical approach to these conditions and the genetic counseling for the patients and their families. For this reason, genomic data have been progressively integrated into diagnostic and therapeutic decisions for BMF, especially for rarer forms [12]. ERCC excision repair 6 like 2 (*ERCC6L2*) represents an emerging germline variant recently identified as responsible for BMF syndrome, with relevant clinical implications due to the elevated risk of progression to MDS/AML [13]. Very limited data are available in the literature so far characterizing biological insights and clinical outcomes of patients and families affected by these germline variants. The aim of this review is to summarize the molecular properties of *ERCC6L2* and the association between germline ERCC6L2 mutations and hematological diseases. We will also describe the phenotypical picture of ERCC6L2-mutated BMF and the potential clinical impact of this condition for hematologists, particularly regarding its management.

## Molecular structure and function of ERCC6L2

The *ERCC6L2* gene is located on chromosome 9 (9q 22.32) and consists of 19 exons (GRCh38.p13). It encodes different helicase-like protein members of the Snf2 family, also including ERCC6, involved in transcription-coupled nucleotide excision repair, and ERCC6L, which has a role in spindle assembly checkpoint [14]. The most described ERCC6L2 isoform is a 712-amino acid protein with a predicted molecular weight of 81 kDa. It structurally presents an N-terminal DEAH ATP-helicase domain and a catalytic helicase C-terminal domain [13]. A 1561-amino acid protein has been also identified, which differs from the ERCC6L2 short isoform by the replacement of the last V712 residue with a new 850-aa peptide. Like the ERCC6L2 short isoform, the long one contains an N-terminal Tudor domain followed by a helicase/ATPase domain, with in addition a C-terminal domain, named HEBO [15]. The expression of *ERCC6L2* is ubiquitous and there is no evidence of tissue-specific regulation of the splicing mechanisms leading to the production of one of the two isoforms [15]. ERCC6L2 acts as ATP-dependent DNA translocase, playing a role in DNA repair, recombination, translocation, and chromatin modeling [16]. Through an integrating CRISPR knockout and chemical perturbation screening approach, ERCC6L2 has proven to play a vital role in the non-homologous end-joining (NHEJ) DNA repair mechanism. Loss of ERCC6L2 led to sensitivity to etoposide and bleomycin as well as a marked reduction in end-joining, confirming the role of ERCC6L2 in promoting canonical NHEJ and in class switch recombination (CSR) of lymphocyte immunoglobulin heavy chain genes [17]. ERCC6L2 knockout cell lines/lymphocytes showed reduced numbers of IgA+ cells following induction of switching, which is highly impaired in class switching suggesting the role of ERCC6L2 in the repair of DNA breaks. Via its C-terminal domain, ERCC6L2 interacts with the XLF end-joining factor and acts in V(D)J recombination. ERCC6L2 controls orientation-specific joining of broken ends and facilitates programmed recombination through directional repair of distant breaks [17]. Thanks to the analysis of ERCC6L2 interactome, through affinity-based mass spectrometry, ERCC6L2 was found to interact not only with DNA repair proteins but also with RNA binding proteins, involved in mRNA elongation and export. ERCC6L2 is specifically able to bind the DNA-dependent protein kinase (DNA-PK), a regulatory member of the RNA Pol II transcription complex, helping in resolving DNA-RNA hybrid structures and minimizing transcription-associated genome instability [18].

## Germline ERCC6L2 mutations and disease

**Fig. 1:**
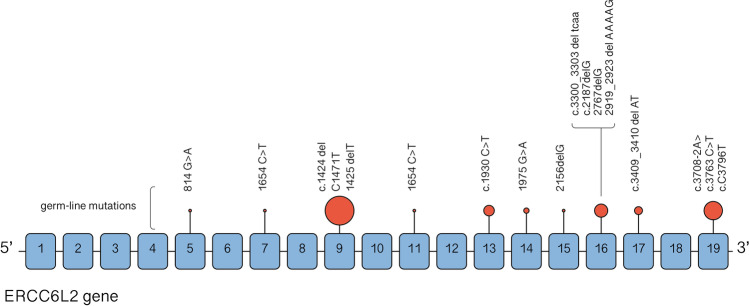
Reported germline homozygous ERCC6L2 mutations. Red circles’ dimension indicates the relative prevalence of mutations

About a decade ago, homozygous germline loss-of-function ERCC6L2 mutations were first described in two patients with BMF syndrome associated with developmental delay and microcephaly [13]. Since then, similar variants have been reported in 31 patients with hematological manifestations, typically presenting with BMF characterized by a high risk of MDS and AML development. Recent reports suggest that these variants could be detected in 3–5% of pediatric and young adult patients with a history of an inherited myeloid disease [4, 19]. Furthermore, an ERCC6L2 variant, namely, c.1424del, was found to be enriched in the Finnish population, suggesting the existence of a founder effect, and was specifically associated with M6 AML, a particularly aggressive [20]. All these findings highlight the emerging role of ERCC6L2 in germline predisposition to myeloid disease, while the functional association between mutation and the hematological manifestations needs to be completely understood. The germline alterations reported in the 31 patients described so far include frameshift and homozygous non-sense mutations affecting both the ERCC6L2 short isoform and the ERCC6L2 long isoform [4]. Reported mutations are summarized in Fig. 1 and listed in Supplementary Table 1. All these *ERCC6L2* mutations cause the introduction of an early stop codon generating a truncated and non-functional protein. ERCC6L2 truncated forms have been demonstrated to display an aberrant localization and conformation, being retained in the endoplasmic reticulum (ER), before degradation [13]. Regarding the potential functional effect, a recent study demonstrated that in vitro models, ERCC6L2 mutation has been shown to produce a significant impairment of the clonogenic potential of hematopoietic stem cells, particularly affecting the erythroid lineage and ultimately resulting in delayed erythropoiesis. The authors also analyzed the molecular pathways affected by ERCC6L2 deficiency showing that DNA repair and TP53 activity pathways were significantly upregulated while genes involved in hematopoietic differentiation were conversely suppressed. Furthermore, this study included the first investigation into the consequences of ERCC6L2 alterations on the stromal niche, revealing in enhanced osteogenesis and suppressed adipogenesis [21]. This microenvironment resembles what was founded in sporadic cases of AML, suggesting the existence of a BM niche particularly prone to malignant transformations in ERCC6L2 mutated patients [22].

## Clinical features of ERCC6L2-mutated disease

### Hematological features

As previously mentioned, thirty-one patients with homozygous germline ERCC6L2 mutations have been reported so far. A description of the studies reporting the cases is summarized in Table 1. Recurrent clinical and biological characteristics of reported patients are shown in Table 2 while detailed information for single patients can be found in Supplementary Table 2. All 31 individuals with homozygous ERCC6L2 germline mutations showed hematological features (except for one in the study by Jarviaho et al., coincidentally identified when screened for possible sibling donor [23]). 13/31 (42%) of patients were male. Most patients presented with trilineage cytopenia: thrombocytopenia (with values at presentation ranging between 4000 and [23] 166,000/μL), anemia (between 7 and 12.7 g/dL), and neutropenia (between 100 and 1600/μL). The median age for referral to medical attention was 19. Regarding the available BM morphology (BM morphology not reported in 5 cases), 20 patients presented a hypocellular BM without dysplastic features while 6 presented dysplasia at the time of presentation. The clinical BMF phenotype was generally mild, with only one patient transplanted because of transfusion dependency and another reported to suffer from fluctuating but severe BMF, in the absence of dysplastic/leukemic progression. Seven patients presented or developed MDS. Four out of these 7 subsequently progressed to AML. Other 3 AML carrying ERCC6L2 mutations were identified [20]. In summary, approximately a third of the whole cohort of patients (10/31) developed MDS or AML. The median age at development of MDS/AML was 31 while considering only leukemic progression, the median age was 49 years old. Notably, not all the patients reached an adequate follow-up able to exclude the progression (the median age at the end of the follow-up was 39), possibly suggesting a higher dysplastic/leukemic penetrance of this disease. Data regarding the time of development of the hematological disorder seems to be consistent with other DNA repair mutations, in contrast to other genes such as GATA2, SAMD9, or SAMD9L in which the progression seems to be earlier, or DDX41 in which it occurs in the elderly [2, 19]. Four of the 10 patients who developed MDS or AML presented the evidence of hypocellularity or anemia before the diagnosis of MDS/AML. Of note, in 7/10 patients progressing to MDS/AML, TP53 mutated clones were described, whereas no such alterations were reported in patients without disease progression. TP53 mutations were loss-of-function and the details of the reported somatic mutations are presented in Supplementary Table 1. TP53 mutations were present with a variant allele frequency (VAF) below 50%, suggesting the presence of heterozygous mutation, but no data on the clones’ trajectory was available. Somatic mutation of TP53 is a shared feature of BMF syndrome, as described in Diamond-Blackfan anemia (DBA), Shwachman-Diamond syndrome (SDS), or in short telomere syndrome. However, the clinical and biological significance differs among the different BMF syndromes, as for SDS which heterozygous TP53 mutations in SDS can persist for years without progression to malignancy. In the case of ERCC6L2-related malignancies, the presence of clones only in patients with progression may suggest a role of this acquired alteration as an early step toward malignancy. Monosomy 7 was also described in 7/10 patients with MDS/AML. Other genomic aberrations and gene variants are reported in Supplementary Table 2. All but one [4] ERCC6L2-related AML were FAB M6. Interestingly, in this regard, Douglas and colleagues analyzed the national Finnish AML registry and found that 4/10 AML M6 cases carried the homozygous ERCC6L2 mutation, in comparison with 0/165 in other FAB AML of the same registry. The median age at diagnosis of AML M6 in ERCC6L2-mutated patients in the registry was 49 compared to 67 in other AML M6 patients. Regarding the clinical outcome, 8/9 patients with MDS/AML died (no outcome data was reported in one patient with MDS), suggesting a dismal prognosis of MDS/AML harboring ERCC6L2 mutations. All patients (7/7) with ERCC6L2-mutated AML died. Hematopoietic stem cell transplantation was performed in 8 cases. Three patients were transplanted before the development of MDS/AML and all survived. Two patients with MDS received transplantation and one of them died of TRM (EBV-related lymphoma) (no outcome data for the second one). Of the three transplanted patients with AML, no one survived due to the relapse of the disease after HSCT, highlighting the aggressiveness of ERCC6L2-mutated leukemia. These significantly poor data on outcomes should be confirmed in larger cohorts. A recent abstract presented at the American Society of Hematology Congress in 2021 reported preliminary comprehensive data on 46 subjects from 31 families with biallelic germline ERCC6L2 variants from across different countries. These data seem to confirm a high penetrance of AML (9/46), predominantly M6, with a median age of leukemic progression of 37 years. All patients with AML showed a complex karyotype with TP53 mutations and a dismal prognosis (all died within 1 year from the diagnosis) [24]. Definitive results of this multinational study are expected to better define the phenotype of this condition, particularly regarding the risk of clonal evolution.

**Table 1:** Main characteristics of the included studies

Authors	Year	*N* of patients	Study design
Tummala et al.	2014	2	WES on 3 genetically uncharacterized cases of BMF
Zhang et al.	2016	1	WES on a genetically uncharacterized case of BMF
Jarviaho et al.	2017	3	WES on 2 genetically uncharacterized cases of BMF and on one of their relatives studied as a possible HSCT donor
Shabanova et al.	2018	1	WES on a genetically uncharacterized case of BMF
Bluteau et al.	2018	7	WES on 179 genetically uncharacterized cases of BMF
Tummala et al.	2018	8	Combination of WES and candidate gene sequencing on genetically uncharacterized cases of BMF and their families
Douglas et al.	2019	8	WES or capillary sequencing in 3 study families and on a validation cohort of 7 cases of AML M6 from the Finnish Hematology Registry
Thams et al.	2020	1	Whole genome sequencing on a patient with congenital mirror movements

**Table 2:** Main hematological features of the reported patients

Male	13/31 (42%)
Mean age at presentation	19 years old (2–65)
Thrombocytopenia (≤150,000/μL)	20/21 (N/A 10)
Leucopenia (≤4500/μL)/neutropenia (≤1500/μL)	17/21 (N/A 10)
Anemia (≤12 g/dL)	17/21 (N/A 10)
Microcephaly/developmental delay	6/31 (19%)
Short telomeres	4/17 (N/A for 16)
Hypocellular BM	20/30
Development of MDS/AML	10/31 (32%)
Mean age at development of MDS/AML	31.5 years old (2–65)
HSCT	8/31
HSCT performed in BMF	3/10
HSCT performed in MDS	2/10
HSCT performed in AML	3/10
Unfavorable outcome (death)	8/30 (N/A for 1)
Unfavorable outcome (death) in patients who underwent HSCT as BMF	0/3
Unfavorable outcome (death) in patients who underwent HSCT as MDS	1/2 (TRM in 1; N/A for 1)
Unfavorable outcome (death) in patients who underwent HSCT as AML	3/3 (100%)

### Focus on non-hematological manifestations

Regarding non-hematological clinical features, results are not uniform among different studies. Regarding phenotypical features, Shabanova and colleagues reported a patient with low-set prominent ears, a pointed prominent chin, deep-set eyes, and one cafè au lait spot [25]. Tummala and colleagues reported a patient with failure to thrive, thin teeth, muscle pain, delayed switch to adult teeth, arterio-venous malformation, café au lait pigmentation, leucoplakia, low birth weight, and short stature were also reported [18]. Somatic features are not reported in other manuscripts and thus it is difficult its attribute to ERCC6L2. Regarding neurological features, 6/31 patients presented microcephaly. Developmental delay and/or learning difficulties were also described in 6 patients and concurrent microcephaly was present in 5 patients [13, 15]. Shabanova and colleagues reported a patient with other neurological and ocular features such as ataxia, dysmetria, nystagmus, rod, and cone dystrophy [25]. Bluteau and colleagues reported the case of a patient with peculiar neurological symptoms, namely, intellectual disability and vascular abnormalities in the right frontal lobe at MRI [4]. Recently, Thams and colleagues reported the presence of homozygous germline ERCC6L2 variant in a patient with congenital mirror movements (CMM) and concomitant MDS. Considering the initial reports, the existence of a neurological phenotype of ERCC6L2 syndrome was suggested [13, 25]; however, recent larger reports seem to exclude the presence of these features within the syndrome’s clinical spectrum [4, 20, 24]. Further studies are needed to define the exact neurological and somatic involvement of the ERCC6L2 mutations.

## Management of ERCC6L2-related disorders

**Fig. 2:**
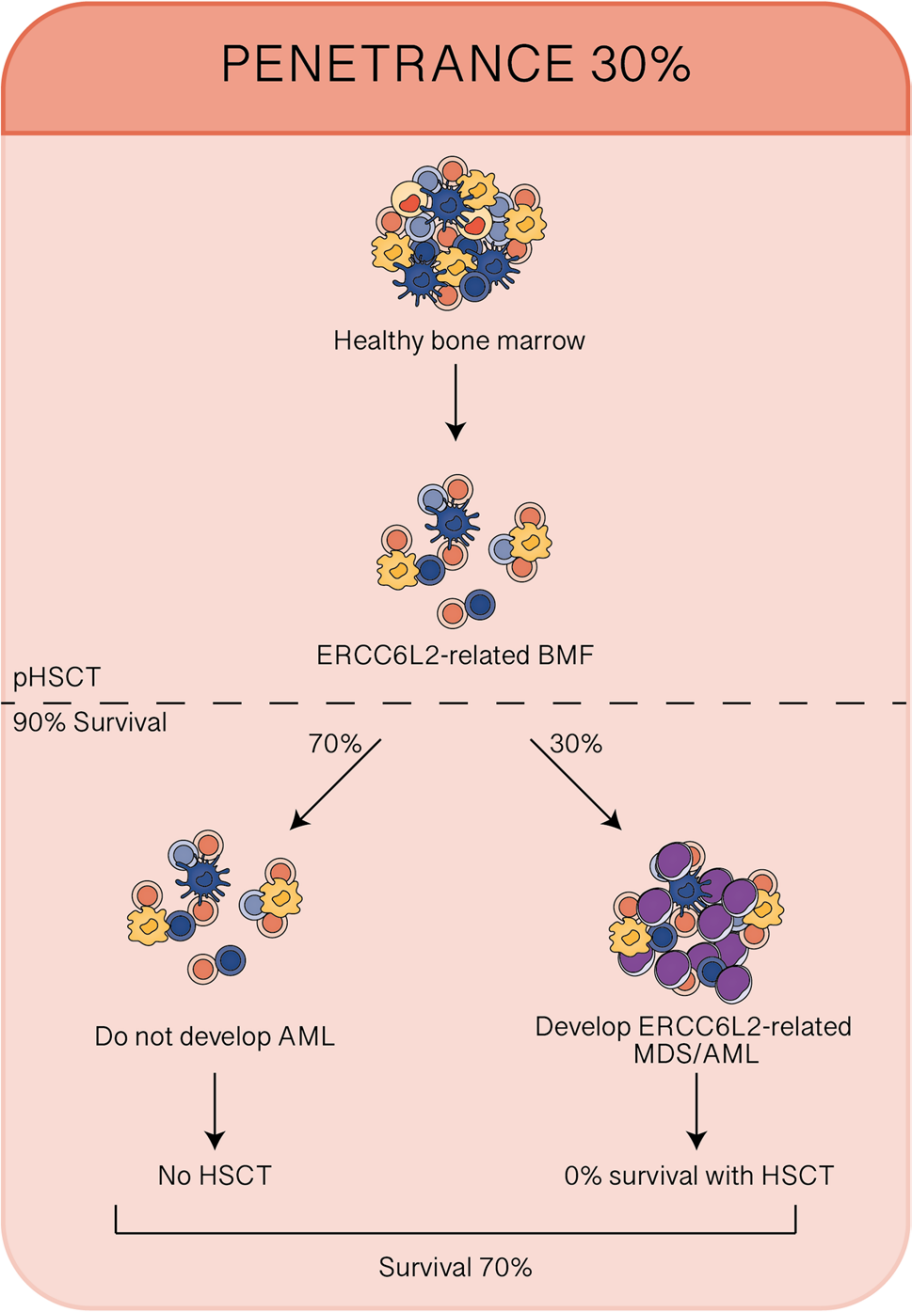
Role of preemptive HSCT in ERCC6L2-related BMF. AML acute myeloid leukemia, BMF bone marrow failure, HSCT hematopoietic stem cell transplantation, pHSCT preemptive HSCT

As mentioned before, the emergence of novel germline variants poses new diagnostic and therapeutic challenges for physicians. In fact, different driving mutations define different clinical pictures which must be fully understood. ERCC6L2-related disorder represents a clinical conundrum due to the scarcity of available evidence and the lack of long follow-up. Considering the high clinical impact and the dismal prognosis of ERCC6L2-mutated AML in the published cohorts, ERCC6L2 should be included in the initial assessment for both BFM and MDS/AML. Clinical features such as age at presentations or the presence of neurological symptoms seem to have a limited role in supporting the diagnosis considering their variability. Early detection of ERCC6L2 mutations is of key importance in preventing inappropriate administration of immunosuppressive therapy. It is also essential in order to guide the right selection of healthy sibling donors in case of indication for HSCT and for adapting the conditioning regimen to avoid toxicity arising from underlying genetic defects [4]. ERCC6L mutation has indeed a high risk of clonal evolution and leukemic progression; thus, a careful follow-up should be provided, as suggested also by Douglas et al. [20, 25]. HSCT should be certainly considered in case of disease progression and transfusion dependency. On the other side, considering the extremely high risk of leukemia progression suggested by the presented cases and the dismal prognosis of patients when MDS and especially AML have developed, the choice of an HSCT before disease progression should be considered, as outlined in Fig. 2 [4, 20, 26]. Certainly, this possibility needs to be confirmed by larger cohort studies with longer follow-up to figure out the exact prevalence of disease progression. In fact, considering that patients with BMF not developing MDS/AML present a general mild clinical phenotype with scarce transfusion need, the risk-benefit assessment should be precisely fine-tuned. In the case of indication for HSCT, the choice of a donor can be particularly challenging, considering the familiar transmission of this condition and the scarcity of data regarding ERCC6L2 mutation carriers. If available, unrelated matched donors should be preferred. Interestingly, preliminary results from Spanish Group of Myelodysplastic Syndrome (GESMD) showed a high rate of heterozygous ERCC6L2 mutations in a cohort of adult MDS suggesting a potential role for heterozygous configuration in MDS onset [27]. Unfortunately, data about transplant procedures performed in these patients are lacking, including donor choice and conditioning regimens adopted. In this regard, more precise results will be provided of the mentioned international ongoing study. The development of TP53 and monosomy 7 have been described in patients that developed MDS and AML and no cases of somatic genetic rescue have been reported [2, 24]. As previously mentioned, the clinical significance of such evidence needs to be defined [28]. Available data suggest including the sequencing for somatic mutations including TP53 and VAF monitoring into clinical surveillance strategy, in order to identify patients with a high risk of leukemic progression and potentially enable preemptive strategies, such as transplantation [29, 30]. Future studies including genetic and functional characterization of these somatic mutations could possibly determine the mechanism of clonal evolution in ERCC6L2-related disorder.

## Conclusions

Germline homozygous ERCC6L2 mutations represent a novel genetic abnormality predisposing for BMF in children and in adults. The high prevalence of progression toward MDS/AML poses several questions in clinical management. Indeed, an early HSCT may be the right choice in the presence of a suitable donor to prevent disease progression, particularly if somatic TP53 mutations occurred. Further studies are needed to elucidate the exact prevalence of progression, the clinical and genetic findings associated with clonal evolution, and the clinical characteristics of the heterozygous status to provide the proper therapeutical management.

## Supplementary information

Supplementary file 1 Supplementary Table 1

Supplementary file 2 Supplementary Table 2

## References

[1] Kennedy AL, Shimamura A (2019). Genetic predisposition to MDS: clinical features and clonal evolution. Blood.

[2] Sahoo SS, Pastor VB, Goodings C (2021). Clinical evolution, genetic landscape and trajectories of clonal hematopoiesis in SAMD9/SAMD9L syndromes. Nat Med.

[3] Bruzzese A, Leardini D, Masetti R (2020). GATA2 related conditions and predisposition to pediatric myelodysplastic syndromes. Cancers (Basel).

[4] Bluteau O, Sebert M, Leblanc T (2018). A landscape of germ line mutations in a cohort of inherited bone marrow failure patients. Blood.

[5] Fabozzi F, Mastronuzzi A, Ceglie G (2022). GATA 2 deficiency: focus on immune system impairment. Front Immunol.

[6] Leardini D, Messelodi D, Muratore E (2022). Role of CBL mutations in cancer and non-malignant phenotype. Cancers (Basel).

[7] McReynolds LJ, Calvo KR, Holland SM (2018). Germline GATA2 mutation and bone marrow failure. Hematol Oncol Clin North Am.

[8] Baccelli F, Leardini D, Muratore E (2022). Immune dysregulation associated with co-occurring germline CBL and SH2B3 variants. Hum Genomics.

[9] Tsai FD, Lindsley RC (2020). Clonal hematopoiesis in the inherited bone marrow failure syndromes. Blood.

[10] Ghemlas I, Li H, Zlateska B (2015). Improving diagnostic precision, care and syndrome definitions using comprehensive next-generation sequencing for the inherited bone marrow failure syndromes. J Med Genet.

[11] Skibenes ST, Clausen I, Raaschou-Jensen K (2021). Next-generation sequencing in hypoplastic bone marrow failure: what difference does it make?. Eur J Haematol.

[12] Sasada K, Yamamoto N, Masuda H (2018). Inter-observer variance and the need for standardization in the morphological classification of myelodysplastic syndrome. Leuk Res.

[13] Tummala H, Kirwan M, Walne AJ (2014). ERCC6L2 mutations link a distinct bone-marrow-failure syndrome to DNA repair and mitochondrial function. Am J Hum Genet.

[14] Shang Y, Long F (2021). Repair of programmed DNA lesions in antibody class switch recombination: common and unique features. Genome Instab Dis.

[15] Zhang S, Pondarre C, Pennarun G (2016). A nonsense mutation in the DNA repair factor Hebo causes mild bone marrow failure and microcephaly. J Exp Med.

[16] Flaus A, Martin DMA, Barton GJ, Owen-Hughes T (2006). Identification of multiple distinct Snf2 subfamilies with conserved structural motifs. Nucleic Acids Res.

[17] Liu X, Liu T, Shang Y (2020). ERCC6L2 promotes DNA orientation-specific recombination in mammalian cells. Cell Res.

[18] Tummala H, Dokal AD, Walne A (2018). Genome instability is a consequence of transcription deficiency in patients with bone marrow failure harboring biallelic ERCC6L2 variants. Proc Natl Acad Sci U S A.

[19] Feurstein S, Churpek JE, Walsh T (2021). Germline variants drive myelodysplastic syndrome in young adults. Leukemia.

[20] Douglas SPM, Siipola P, Kovanen PE (2019). ERCC6L2 defines a novel entity within inherited acute myeloid leukemia. Blood.

[21] Boyd AL, Reid JC, Salci KR (2017). Acute myeloid leukaemia disrupts endogenous myelo-erythropoiesis by compromising the adipocyte bone marrow niche. Nat Cell Biol.

[22] Armes H, Bewicke-Copley F, Rio-Machin A (2022). Germline ERCC excision repair 6 like 2 (ERCC6L2) mutations lead to impaired erythropoiesis and reshaping of the bone marrow microenvironment. Br J Haematol.

[23] Järviaho T, Halt K, Hirvikoski P (2018). Bone marrow failure syndrome caused by homozygous frameshift mutation in the ERCC6L2 gene. Clin Genet.

[24] Hakkarainen M, Douglas SPM, Vulliamy T (2021). Multinational study on the clinical and genetic features of the ERCC6L2-disease. Blood.

[25] Shabanova I, Cohen E, Cada M (2018). ERCC6L2-associated inherited bone marrow failure syndrome. Mol Genet Genomic Med.

[26] Dini G, Zecca M, Balduzzi A (2011). No difference in outcome between children and adolescents transplanted for acute lymphoblastic leukemia in second remission. Blood.

[27] Carrillo-Tornel S, Chen-Liang TH, Yeguas Bermejo A (2022). ERCC6L2 in early-onset adult myelodysplastic syndrome without pre-existing disorder. Blood.

[28] Desai P, Mencia-Trinchant N, Savenkov O (2018). Somatic mutations precede acute myeloid leukemia years before diagnosis. Nat Med.

[29] Kennedy AL, Myers KC, Bowman J (2021). Distinct genetic pathways define pre-malignant versus compensatory clonal hematopoiesis in Shwachman-Diamond syndrome. Nat Commun.

[30] Cesaro S, Donadieu J, Cipolli M (2022). Stem cell transplantation in patients affected by Shwachman-Diamond syndrome: expert consensus and recommendations from the EBMT Severe Aplastic Anaemia Working Party. Transplant Cell Ther.

