# Supplementary material for: ERCC6L2-related disease: a novel entity of bone marrow failure disorder with high risk of clonal evolution
Source: Ann Hematol. 2023 Feb 15;102(4):699–705. doi: 10.1007/s00277-023-05128-2 (PMC9998559; doi:10.1007/s00277-023-05128-2)
Supplement: Supplementary file 1 — Supplementary Table 1 [file 277_2023_5128_MOESM1_ESM.docx]

|  | Mutation | Exon | Somatic TP53 status, VAF | Reference |  |
| --- | --- | --- | --- | --- | --- |
| Case 1 | c.1930 C > T | 13 | N/A | (Tummala et al. 2014) |  |
| Case 2 | c.1203 > 1206 | 7 | N/A | (Tummala et al. 2014) |  |
| Case 3 | c.1930 C > T | 13 | N/A | (Zhang et al. 2016) |  |
| Case 4 | c. 1424 del | 9 | N/A | (Järviaho et al. 2018) |  |
| Case 5 | c. 1424 del | 9 | N/A |  |  |
| CASE 6 | c. 1424 del | 9 | N/A |  |  |
| Case 7 | 1654 c > T | 11 | N/A | (Shabanova et al. 2018) |  |
| Case 8 | c.2187delG c.3708-2A> | 16  19 | N/A | (Bluteau et al. 2018) |  |
| Case 9 | c.2187delG c.3708-2A> | 16  19 | N/A |  |  |
| Case 10 | c.C1471T  c.C3796T | 9  19 | N/A |  |  |
| Case 11 | c.C1471T  c.C3796T | 9  19 | N/A |  |  |
| Case 12 | c.1930 C > T | 13 | N/A |  |  |
| Case 13 | 814 G>A | 5 | N/A |  |  |
| Case 14 | c.1930 C > T | 13 | N/A |  |  |
| Case 15 | 2767delG | 16 | N/A | (Tummala et al. 2018) |  |
| Case 16 | 1975 G>A | 14 | N/A |  |  |
| Case 17 | 1975 G>A | 14 | N/A |  |  |
| Case 18 | 2156 delG  c.3300_3303 del tcaa | 15  16 | N/A |  |  |
| Case 19 | c.3409_3410 del AT  c.3763 C>T | 17  19 | N/A |  |  |
| Case 20 | c.3409_3410 del AT  c.3763 C>T | 17  19 | N/A |  |  |
| Case 21 | c.3409_3410 del AT  c.3763 C>T | 17  19 | N/A |  |  |
| Case 22 | 2919_2923 del AAAAG | 16 | N/A |  |  |
| Case 23 | 1425 delT | 9 | c.532C>G, 35% | (Douglas et al. 2019) |  |
| Case 24 | 1425 delT | 9 | c.517G>A, N/A |  |  |
| Case 25 | 1425 delT | 9 | None |  |  |
| Case 26 | 1425 delT | 9 | c.577C>T, N/A c.818G>A, N/A |  |  |
| Case 27 | N/A Biological material |  | N/A |  |  |
| Case 28 | 1425 delT | 9 | c.743G>A, 5% c.830G>T, 23% c.843C>A, 11% |  |  |
| Case 29 | 1425 delT | 9 | c.659A>G, 31% |  |  |
| Case 30 | 1425 delT | 9 | c.818G>A, N/A c.856G>A, N/A |  |  |
| Case 31 | N/A | 11 | N/A | (Thams et al. 2020) |  |
