# Supplementary material for: ERCC6L2-related disease: a novel entity of bone marrow failure disorder with high risk of clonal evolution
Source: Ann Hematol. 2023 Feb 15;102(4):699–705. doi: 10.1007/s00277-023-05128-2 (PMC9998559; doi:10.1007/s00277-023-05128-2)
Supplement: Supplementary file 2 — Supplementary Table 2 [file 277_2023_5128_MOESM2_ESM.docx]

|  | Case 1 (Tummala et al. 2014) | Case 2 (Tummala et al. 2014) | Case 3 (Zhang) | Case 4 (Jarviaho et al.) | Case 5 (Jarviaho et al.) | Case 6 (Jarviaho et al.)  Brother of case 5 | Case 7 (Shabanova) | Case 8 (Bluteau) | Case 9 (Bluteau)  Sister of case 8 | Case 10 (Bluteau) | Case 11 (Bluteau)  Sister of case 10 | Case 12 (Bluteau) |
| --- | --- | --- | --- | --- | --- | --- | --- | --- | --- | --- | --- | --- |
| ERCC6L2 mutation | c.1963 C>T | c.1236­_1239delAACA | c.1963 C>T | c.1457del | c.1457del | c.1457del | c.1687C>T | c.2187delG c.3708-2A>T | c.2187delG c.3708-2A>T | c.C1504T  c.C3796T | c.C1504T  c.C3796T | c.C1963T |
| Gender | M | F | M | M | F | M | F | M | F | F | F | M |
| Age at presentation | 12 | 19 | 13 | 8 | 8 | 9 | 7 | 7 | 13 | 22 | 18 | 2 |
| Platelet counts (x10^9^/L) | 9 | 33 | 4 | 5 | 23 | 97 | 58 | 64 | <150 | 107 | 101 | 48 |
| Hemoglobin (g/dL) | 9.9 | 9.3 | 9.0 | 8.1 | 10.6 | Normal | 13.3 | 11.4 | <12 | 11.9 | 12.9 | 10.9 |
| WBC (x10^9^/L) | 2.4 | 5.3 | ANC 0.7 | 2.3 | 2.0 | 4.3 | 3.0 | ANC <1.5 | ANC <1.5 | ANC 0.4 | ANC 1.6 | ANC 1 |
| Bone marrow cellularity | Hypocellular | Hypocellular | Hypocellular | Hypocellular | Hypocellular | N/A | Hypocellular | Hypocellular | Hypocellular | Hypocellular, dysplasia at diagnosis | N/A | Hypocellular |
| Developmental delay and/or learning difficulties | Yes | Yes | Yes | No | No | No | Yes | No | No | No | No | No |
| Microcephaly | Yes | Yes | Yes | No | No | No | Yes | No | No | No | No | No |
| Other clinical features & family history | Facial dysmorphia and ear abnormalities | Floppy during infancy | Bilateral pyeloureteral junction abnormalities; <1SD for weight and <0,5SD for height; vascular abnormalities in right frontal lobe | Height and weight <2.7SD; vitamin B12 deficiency and pubertal delay | None | None | Hypertonia; ataxia, dysmetria, nystagmus; rod and cone dystrophy; low set prominent ears, pointed prominent chin, deep-set eyes | None | None | None | None | Facial dysmorphia |
| Chromosomal breakage with and without MMC and DEB | Normal | Normal | Normal | N/A | N/A | N/A | Normal | N/A | N/A | N/A | N/A | N/A |
| Telomere length | Normal | Short | Normal | Normal | Normal | Normal | Short | N/A | N/A | N/A | N/A | N/A |
| BM karyotype | N/A | N/A | N/A | N/A | N/A | N/A | N/A | 46,XX | 46,XX | - 7 | N/A | 46, XY |
| Dysplasia/AML | No | No | No | No | No | N/A | No | No | No | Yes (MDS at 22 years old) | No | No |
| HSCT | No | No | No | Yes (due to platelet and RBC dependency) | No | N/A | No | Yes | Yes | Yes | No | No |
| Outcome | N/A | N/A | CBC parameters improved spontaneously and stabilized; macrocytosis, moderate neutropenia and thrombocytopenia | Alive | Alive (transfusion-independent) | Coincidentally discovered to carry the mutation when examined as a possible sibling donor | Alive (stable) | Alive | Alive | Died at 24y (TRM, EBV lymphoma post HSCT) | Alive (stable thrombo cytopenia and neutropenia) | Mild thrombo  Cytopenia |
| Length of follow up a | N/A | N/A | 20 years old | 17 years old | 12 years old | N/A | 10 years old | 15 years old | 27 years old | 24 years old | 26 years old | 15 years old |

|  | Case 13 (Bluteau et al.) | Case 14 (Bluteau et al.) | Case 15 (Tummala et al. 2018) | Case 16 (Tummala et al. 2018) | Case 17 (Tummala et al. 2018)  Sister of case 16 | Case 18 (Tummala et al. 2018) | Case 19 (Tummala et al. 2018) | Case 20 (Tummala et al. 2018)  Brother of 19 and 21 | Case 21 (Tummala et al. 2018)  Brother of 20 and 21 |
| --- | --- | --- | --- | --- | --- | --- | --- | --- | --- |
| ERCC6L2 mutation | c.G847A | c.C1963T | c.2767delG | c.2006G>A | c.2006G>A | c.2189delG  c3333_3336delTCAA | c.3442_3443delAT  c.3796C>T | c.3442_3443delAT  c.3796C>T | c.3442_3443delAT  c.3796C>T |
| Gender | F | M | F | M | F | F | F | M | F |
| Age at presentation | 22 | 13 | 8 | 13 | 3 | 17 | 18 | 2 | 13 |
| Platelet counts (x10^9^/L) | 38 | 4 | N/A | 58 | 131 | 90 | 166 | 12 | 85 |
| Hemoglobin (g/L) | 10.7 | 9 | N/A | 7 | 11.2 | 12 | 11.9 | 9.7 | 12.7 |
| WBC (x10^9^/L) | ANC 0.1 | 0.7 | N/A | 2 | 8.6 | 3.5 | 2.4 | 3 | 3.9 |
| Bone marrow cellularity | Hypocellular, dysplasia at diagnosis | Hypocellular | Hypocellular | Hypocellular | Hypocellular | Hypocellular | Hypocellular | Hypocellular | Hypocellular |
| Developmental delay and/or learning difficulties | No | Learning difficulties, intellectual disability, vascular abnormalities in the right frontal lobe | No | No | No | No | No | No | No |
| Microcephaly | No | Yes | No | No | No | Yes | No | No | No |
| Other clinical features & family history |  | Bilateral pyeloureteral junction abnormalities |  | Failure to thrive, thin teeth, muscle pain |  | Delayed switch to adult teeth |  | AV malformation |  |
| Chromosomal breakage with and without MMC and DEB | N/A | N/A | Normal | Normal | Normal | Normal | Unknown | Normal | Unknown |
| Telomere length | N/A | N/A | Normal | Short | Short | Normal | Normal | Normal | Normal |
| BM karyotype | 46, XX; acquisition of -7 | 46,XY | N/A | N/A | N/A | N/A | N/A | N/A | N/A |
| Dysplasia/AML | Yes (MDS at 22 years old, progressed to AML at 43 years) | No | No | No | No | No | No | Yes (MDS at 2 years old, then progressd to AML) | No |
| HSCT | No | No | N/A | N/A | N/A | N/A | N/A | N/A | N/A |
| Outcome | Died at 43 years old | Stable (macrocytosis without anemia, no neurological signs) | N/A | N/A | N/A | N/A | N/A | Died | N/A |
| Length of follow up | 43 years old | 21 years old | N/A | N/A | N/A | N/A | N/A | N/A | N/A |

|  | Case 22 (Tummala et al. 2018) | Case 23 (Douglas et al.) | Case 24 (Douglas et al.)  Sister of case 23 | Case 25 (Douglas et al.)  Niece of 26, 27, 28 | Case 26 (Douglas et al.)  Sister of 27 and 28, paternal aunt of 25 | Case 27 (Douglas et al.)  Sister of 26 and 28, paternal aunt of 25 | Case 28 (Douglas et al.)  Sister of 26 and 27, paternal aunt of 25 | Case 29 (Douglas et al.) | Case 30 (Douglas et al.) | Case 31 (Thams et al.) |
| --- | --- | --- | --- | --- | --- | --- | --- | --- | --- | --- |
| ERCC6L2 mutation | c.2952_2956delAAAAG | c.1457delT | c.1457delT | c.1457delT | c.1457delT | N/A | c.1457delT | c.1457delT | c.1457delT | Homozygous 10-kb deletion in exon 11 of ERCC6L2 |
| Gender | M | M | F | F | F | F | F | F | M | M |
| Age at presentation | 12 | MDS at 38 | MDS at 36 | 14 | anemia during childhood (BMF?) [AML 56 years] | anemia during childhood (BMF?) [AML 38 years] | 47 | AA at 11; mild cytopenia at 20; BMF at 31 | N/A [AML 65 years] | 21 |
| Platelet counts (x10^9^/L) | 102 | N/A | N/A | N/A | N/A | N/A | N/A | N/A | N/A | N/A |
| Hemoglobin (g/L) | 8.1 | N/A | N/A | N/A | N/A | N/A | N/A | N/A | N/A | N/A |
| WBC (x10^9^/L) | 4.9 | N/A | N/A | N/A | N/A | N/A | N/A | N/A | N/A | N/A |
| Bone marrow cellularity | Hypocellular, dysplasia at diagnosis | MDS with excess of blasts Hypercellularity with erythroid predominance (75%), ring sideroblasts (10%) and 14% of myeloid blasts counted from non-erythroid cells | MDS with 10% of ring sideroblasts | Hypocellular | N/A | N/A | Hypocellular | Hypocellular | N/A | Dysplasia at diagnosis |
| Developmental delay and/or learning difficulties | Yes | N/A | N/A | N/A | N/A | N/A | N/A | N/A | N/A | N/A |
| Microcephaly | No | N/A | N/A | N/A | N/A | N/A | N/A | N/A | N/A | N/A |
| Other clinical features & family history | Cafè au lait pigmentation, leucoplakia, low birth weight, short stature | Father died as a result of CRC in his 50s; paternal cousin died of pancreatic cancer in 40s | Melanoma in situ x2  Father died as a result of CRC in his 50s; paternal cousin died of pancreatic cancer in 40s |  |  |  | Family history of solid tumor and grandma died of leukemia NOS | Central vein thrombosis, Rathke’s cyst  Sister died as a result of AA at 34 | Tubular adenoma with dysplasia in rectum at 59  Sister died of severe aplastic anemia at a young age | Congenital mirror movement |
| Chromosomal breakage with and without MMC and DEB | Normal | N/A | N/A | N/A | N/A | N/A | N/A | N/A | N/A | N/A |
| Telomere length | Normal | N/A | N/A | N/A | N/A | N/A | N/A | N/A | N/A | N/A |
| BM karyotype | -7, -20 | Hypodyploid 41-43, **-5, -7**, -17, -18, -19, -20  Acquired somatic TP53 mut | Hypodyploid 43, **-7, -12, 5q-**  Acquired somatic TP53 mut | Normal | t(3;12;?), t(12;?), -7, -5, t(5;7)  Acquired somatic TP53 mut | Complex | N/A  Somatic TP53 mut | Somatic TP53 mut clone | 42-46, del(5)(q31), dup(5)(q31)/t(5;5), -7, 11q23/MLL amplification or translocation, -4  Somatic TP53 mut | Somatic TP53 variant in 20% of bone marrow cells |
| MDS/AML | Yes (MDS at 12 years) | Yes (MDS at 38 progressed to AML M6 at 39 years) | Yes (MDS at 36, progressed to AML M6 at 37 years) | No | Yes (AML M6 at 56 years) | Yes (AML M6 at 36 years) | No | No | Yes (AML M6 at 65 yers) | Yes (MDS at 21 years) |
| HSCT | N/A | Yes | No | No | Yes | No | No | No | Yes | Yes |
| Outcome | N/A | Died (relapse) | Died (relapse after chemotherapy) | Alive | Died (relapse) | Died (refractory disease) | Alive (mild neutropenia and thrombo cytopenia) | Alive (initial spontaneous recoveryfrom AA; then mild neutropenia and thrombo cytopenia; then severe BMF 20y later) | Died (relapse) | N/A |
| Length of follow up | N/A | N/A | N/A | N/A | / | / | N/A | N/A | / | N/A |
